# Local activity in dendrites controls STDP by altering NMDA receptor kinetics

**DOI:** 10.1186/1471-2202-14-S1-P399

**Published:** 2013-07-08

**Authors:** Rajeev V Rikhye, Xulin Tan, Antonius MJ Van Dongen

**Affiliations:** 1 Department of Brain and Cognitive Sciences, Massachusetts Institute of Technology, Cambridge, MA 02139, USA; 2 Program in Neuroscience and Behavioral Disorders, Duke-NUS Graduate Medical School, Singapore 169857, Singapore

## 

Spike timing-dependent plasticity (STDP) is a temporally asymmetric form of Hebbian plasticity in which the sign and magnitude of synaptic strength is determined by the precise timing between pre- and postsynaptic spikes.

Postsynaptic NMDA receptors (NMDARs) play an important role in STDP due to their characteristic magnesium ion (Mg^2+^) gating process [1]. The Mg^2+ ^gate is opened by near coincident presynaptic glutamate release and postsynaptic spiking. Contrary to previous belief, this gating process is not instantaneous but has distinct fast and slow time constants, which might influence the time window over which spike-EPSP coincidence occurs [2,3]. The aim of this study is to investigate how local activity in dendrites alters NMDAR kinetics and in turn controls the time window over which STDP occurs.

Using a detailed biophysical model of an NR2A subunit, we find the NMDAR current to be temporally asymmetric with a faster block than unblock process. This is due to the voltage-dependent fast and slow time constants of Mg^2+ ^gating. These time constants were on the order of 0.8 ms and 5 ms respectively and changed nonlinearly with membrane potential. By varying both amplitude and duration of a model action potential (AP), we find that dendritic Ca^2+ ^spikes cause Mg^2+ ^unblock to be dominated by its fast time constant. Conversely, somatic Na^+ ^spikes elicit a slower unblocking. Since backpropagating APs (BAPs) attenuate along the dendritic arbor, our results suggest that NMDAR kinetics vary depending on synapse location.

Next, we incorporated this biophysical model into a compartmentalized model of a neocortical pyramidal cell in order to investigate the effect that dendritic inhibition and synapse location has on STDP. NMDAR kinetics is significantly different in the basal and apical compartments, which gives rise to a heterogeneous set of STDP curves along the length of the dendrite. Distal synapses had a broader potentiation window than apical synapses, suggesting that distal branches are less selective to correlated inputs. This synapse-location dependent STDP learning rule agrees with previous findings [4]. Dendritic inhibition plays an important role in shaping the computations performed by pyramidal neurons in the neocortex. However, the role that inhibition plays in controlling synaptic plasticity is less clear. By incorporating inhibitory synapses in our model, we find that varying the timing of inhibition relative to the EPSP shifted the peak of the STDP curve, resulting in a reversal of the temporal order of depression and potentiation. Inhibition altered NMDAR kinetics by increasing its blocking rate. As a result, changing the balance between excitation and inhibition in the dendritic branch results in a sliding threshold between LTP and LTD.

In summary, we show that NMDAR kinetics can be altered in an activity-dependent manner resulting in different STDP learning rules. When taken in context of the BCM theory, our finding implies that dendritic inhibition can dynamically alter the threshold between LTP and LTD by altering the time constants of the NMDAR. Our model provides a novel framework for understanding how STDP in different dendritic compartments selects inputs alters information processing in individual neurons.
